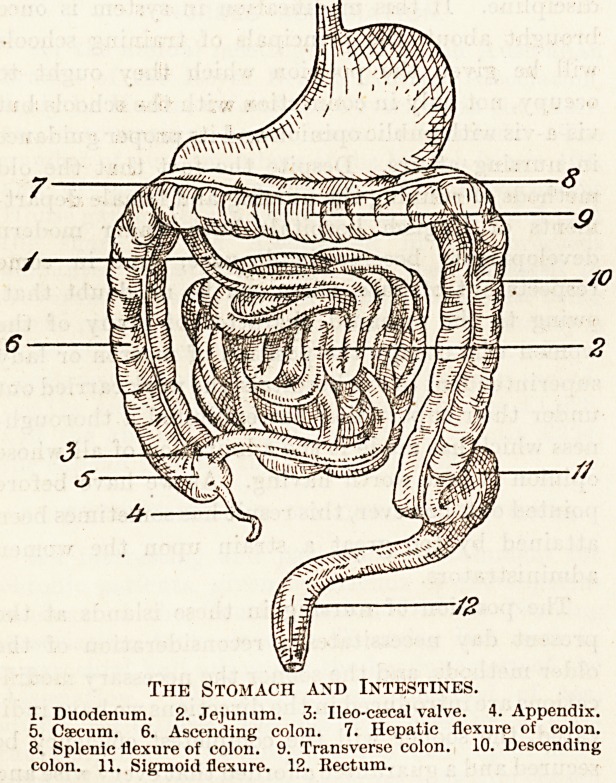# "The Hospital" Nursing Section

**Published:** 1906-06-09

**Authors:** 


### The Hospital.

### Nursing Section.

Contributions for this Section of "The Hospital" should be addressed to the Editor, "The Hospital" Nursing Section, 28 & 29 Southampton Street, Strand, London, W.C.

No. 1,028.—Vol. XL. SATURDAY, JUNE 9, 1906.

### Notes on News from the Nursing World.

### DEATH OF A CRIMEAN NURSE.

One by one the goodly company of the devoted women who served with Miss Nightingale during the Crimean War are passing away. The death took place last week, at Portobello, of Mrs. Margaret Irving, one of the few survivors of this illustrious band. The daughter of a lecturer in Edinburgh University, she was seventy-five years of age, and the widow of Mr. Charles Irving, artist, of Liverpool, a relation to the late Sir Henry Irving. Mrs. Irving was a fully qualified surgical and medical nurse, and possessed the medal for service in the Crimean war. Before she resided in Portobello she was prominently identified with charitable work in Coventry.

### NURSING ON STEAMSHIPS.

The statement is again made that trained nurses are kept on "several of our big steamships." We quite recently pointed out that, while there may be companies which, like the Royal Mail, sometimes engage trained nurses as stewardesses on conditions which are not calculated to attract highly educated women, the only British line of importance which makes a practice of employing trained nurses to undertake ordinary nursing work is the Booth Steamship Company. It is possibly in consequence of the success which has attended their enterprise that the managers of the Hamburg-American line have engaged a trained nurse for their newest ship. But as there is not the slightest prospect of any vacancies on the vessels of the Booth line, English nurses who desire experience of this character will, we fear, have to wait a long time for the opportunity.

### PREMIUMS AT WORCESTER INFIRMARY.

At a meeting of the Executive Committee of Worcester Infirmary the Chairman referred to the fact that the staff of probationers was not at full strength, and said that the engagement of five outside nurses involved a considerable extra expenditure. He stated that the matron had informed him that she thought their terms for probationers were not sufficiently liberal, because she found that, whilst many applications were sent in, few of the applicants proceeded further when they knew the terms. We have no doubt that the matron is right. At Worcester Infirmary the old custom of requiring a premium of £10 from probationers is still observed. This, at any rate, should be abolished for nurses who train for three years, an arrangement which obtains at Salisbury Infirmary. The matter has been referred to the House Committee, and it may be hoped that, as the result of the inquiry, Worcester Infirmary will come into line with similar institutions in the country. The Chairman himself has ascertained that at nearly all of these the practice of imposing a premium has been discontinued.

### NURSES AND NURSING HOMES.

At the sittings of the Palatine Court at Liverpool the other day an application was made to the Vice-Chancellor on behalf of Miss Strange, matron of the St. Annes and Lytham Nursing Institution, for an order to restrain Nurse Harty, lately of the Home staff, from carrying on nursing within 10 miles of St. Annes in breach of her agreement with Miss Strange. Counsel explained that it was necessary to take this step to prevent such agreements being broken. After hearing Nurse Harty, who promised not to repeat her offence, the Court made the usual restraining order against her. The judgment of the Court confirms the statement frequently made by us that engagements similar to that undertaken by Nurse Harty are binding, and we hope that by making the decision widely known we may do something to prevent nurses with elastic consciences from breaking their agreements.

### DIFFICULTIES AT PLYMOUTH INFIRMARY.

Recently the Guardians selected, from applications sent in, a new assistant nurse at Plymouth Workhouse Infirmary. The nurse, however, wrote back asking that her fare should be paid from Landport, explaining that as "she had been taken in several times, and wished to guard against it in future, she never took up an appointment without previously seeing her apartments, the superintendent, and the wards." She suggested that an interview would be beneficial to all parties. The Guardians declined to comply with her request, and it was decided to ask her to state definitely whether she intended to take up the appointment. At the same meeting two nurses attached to the Infirmary tendered their resignations. Their letters were referred to the Hospital Committee, but they were not read in consequence of objection being taken.

### THE NURSE IN THE ALMSHOUSE.

An interesting paper was read at the annual meeting of the Michigan State Nurses' Association, in which the author, having condemned the existing conditions of the inmates of the county almshouses, urged that the latter should be put on the same basis as infirmaries. The inmates, she contended, should be recognised as legitimate subjects for State care, just in the same manner as the blind, the deaf, or the insane. The practical result of the discussion which followed the reading of the paper was the appointment of a committee to confer with the State Federation of Women's Clubs in order to frame plans for inaugurating the movement. This proposal from the other side of the Atlantic for the introduction of trained nurses for the sick poor in almshouses may be worthy of emulation here. No doubt attention is given to inmates of some of our almshouses by district nurses. But others, we fear, are severely let alone, though there will nearly always be persons in such institutions requiring the services which none but a nurse can render. Moreover, the effect of the work upon the nurse herself must be to broaden and intensify her sympathies.

### QUARANTINE FOR PUERPERAL FEVER.

The question of compensation for loss of employment has arisen in connection with a nurse at Hampton. A few weeks ago the Medical Officer of Health advised the nurse, who works under the auspices of the Hampton District Council, to abstain, after the termination of her attendance on a case of puerperal fever, from nursing any lying-in case for eight weeks. She had been engaged on the case for five weeks until recovery and suffered nothing in the form of sores, but the medical officer considered that two months' abstention was not too long a period for safety in dealing with such a dangerous disease. The Local Government Board, however, on application being made by the District Council for sanction to compensate the nurse in consequence of her loss of employment for two months, intimated that the period was unnecessarily long, and referred the matter back. We appreciate the motive for the precautions adopted by the medical officer, and hope that the nurse will not, in any event, suffer in pocket. But the period of quarantine was unduly protracted.

### WORKING MEN AND DISTRICT NURSING ASSOCIATIONS.

The working men of Blackburn have set an admirable example. Out of gratitude for the help given to their families during sickness by the District Nursing Association, they last year came forward and offered to give help to clear off the debt on the Home of £500. Their offer was gladly accepted by the Executive Committee, and they decided to start a 10,000 shilling fund. By means of house-to-house collections they have collected about £200 in shillings. They enlisted the sympathies of the manager of the Palace Theatre, and, as a result of gala matinées, added £180 to the fund. A jumble sale organised amongst themselves realised £30, and assistance from other sources has brought up the sum altogether to £420. The task, they admit, proved far more difficult than they anticipated, but they are confident that, in addition to the money they have raised, the objects and needs of the Association are now far better known than they were before. We are quite sure of this, and we should like to see their action widely emulated.

### PROVIDING FOR A TEMPORARY EMERGENCY.

At the last meeting of the Isle of Wight Guardians there was a long discussion on the infirmary nursing staff, in which Mr. Baldwyn Fleming, the Local Government Board Inspector, took part. It appears that the superintendent-nurse had informed the inspector that the staff on the female side is inadequate at the present time, owing to the wards being quite exceptionally full of helpless cases, and that he himself found two or three cases of bed-sores. It is fair to the Guardians to say that they did not manifest any indisposition to afford assistance, but, taking into consideration the fact that the strain was not likely to be more than temporary, they proposed, and the inspector sanctioned, the appointment of an able-bodied woman from outside, none being available at the workhouse, to help with the lifting. Assuming that if the emergency lasts for a protracted period an extra nurse will be engaged, there is no fault to be found with the arrangement.

### A NEW ASYLUM MATRON.

An excellent appointment was made last week to the matronship of the Baldowan Asylum for Imbecile Children in Forfarshire. Miss A. O. Henry, the successful candidate, has had just the experience qualifying her for the duties. Subsequent to her training at the Glasgow Sick Children's Hospital she spent a year in Broomhill Incurable Hospital, near Glasgow; and after her general training at Dundee Royal Infirmary, she was placed in charge of the children's ward. This position she has held for upwards of twelve years. Her work among the children of her ward has been admirable, and very evidently largely a labour of love, while the assistance she gave to the medical staff showed her to possess the qualities which make a successful nurse and sister. The numerous friends of Miss Henry wish her every happiness in her new sphere, but they cannot help regretting that her promotion means the severance of a tie which has so long and so satisfactorily existed between the Royal Infirmary children's ward and its kindly sister.

### NURSES' MISSIONARY LEAGUE.

Following the morning conference of the Nurses' Missionary League, held on Wednesday, of which we gave an account last week, about 60 guests were received in the afternoon by Mrs. Dougall and Miss Miller, while other members of the committee dispensed hospitality. Dr. Jays, of the North-West African Mission, brought interesting relics to be viewed; charms composed of pieces of cord attached to hideous images some six inches high; and as surgical instruments, a probe six to eight inches, fairly thick and made of iron, and a knife, made also of iron. At the evening meeting the chair was taken by Mr. McAdam Eccles, and Dr. Hill, of China, gave an eloquent address, pleading for missionary nurses. He mentioned that at an abdominal operation he had no nurses, only a native convert to give the anæsthetic. He had to subsequently nurse the patient himself. At another time he had the assistance of a missionary trained nurse, who moved her bed to the hospital and slept by the patient in order to attend to her through the night, besides taking all her other varied duties throughout the day. Miss Perry, who has recently returned from Korea, said that the custom there is to put the typhoid patients on to the walls, and leave them to die; these walls being about ten feet wide, and when walking about it was quite a common thing to come across funereal-looking heaps, which were moribund human beings. Miss Perry was attired in native indoor dress, whilst Miss Pash, another missionary, wore the Korean outdoor garb, which somewhat resembles a Japanese kimono.

### SOUTH AUSTRALIAN BRANCH OF THE BRITISH NURSES' ASSOCIATION.

At the annual meeting of the South Australian branch of the Royal British Nurses' Association, Lady Le Hunte, wife of the Governor, in the chair, an encouraging report was submitted. The number of nurse members of the branch is now 101, and of medical members 31. The Government House garden fête in October was so successful that the share of the Association in the proceeds amounted to £1,000, which has been invested in Government bonds for four years, the interest being at the rate of 4 per cent. per annum. This money is to be held in reserve for the purpose of securing a permanent home. The receipts for the year, including the amount realised from the fête, was £1,228, and the amount brought forward to credit £224, the expenditure for the year being £325.

### AN ENTERTAINMENT TO CHRONIC PATIENTS.

An interesting and pathetic entertainment for chronic patients, given by friends of the superintendent of the South London District Nursing Association, took place on Thursday last week in a parochial hall adjoining the headquarters of the Association. The programme consisted of tea, music, recitations, and performances by a first-rate conjuror. The audience was made up of more than 200 chronic patients, each nurse having chosen about twenty out of her own particular district, the area of the Association covering Waterloo, Vauxhall, Lambeth, Clapham, Battersea, and parts of Balham and Wandsworth. The sight of the emaciated, strained faces, with their various expressions, all turned eagerly towards the stage, was one seldom witnessed. First came a row of spinal carriages, occupied by patients of various ages, from 3½ to 40 years; next Bath chairs with older men and women, several very old; and after this, row upon row of more active patients, of all ages, some with heads bound up, others with arms in slings, crutches, eyes bandaged. There were even blind and deaf people amongst the audience, and all with the same expression of real enjoyment. One very old man, to whom his nurse was administering a morphia injection before his journey home, when asked if he were not very tired replied, "Oh! ma'am, I'll get over that; but 'tis the kind faces, and good cheer, and the heavenly charity of it all, that's what'll last." The wives of several of the neighbouring clergy and medical men were asked by the nurses to help dispense the tea; and, judging from the uninterrupted way in which the proceedings were conducted, they must have responded very heartily. The enthusiastic response to the proposal of Miss Bullock, the superintendent, that three cheers should be given for Mr. Boyle and the kind friends who had come from so far to give them such a delightful afternoon, left no doubt as to the feeling of the guests.

### ST. PANCRAS SOUTH INFIRMARY.

The result of the examination of probationers at the St. Pancras South Infirmary is highly creditable to the medical superintendent, the matron, and themselves. Mr. Rupert Bucknall, of Harley Street, who conducted the examination, reports that the 14 probationers who presented themselves all passed, four obtaining more than 90 per cent. of the maximum of marks. Special mention was made of Nurse Rolfe, who, according to the examiner, passed with very great distinction. The examiner says that he does not remember to have met with such excellent examination results, and that the nurses "showed a splendid all-round knowledge of their work."

### HACKNEY POOR-LAW INFIRMARY.

In his report on the examination of probationers at Hackney Poor-law Infirmary, Dr. F. J. Smith, the examiner, congratulates the medical staff and the matron on the "very evident pains and care they had taken in the teaching, as judged by the more than excellent results." Out of 14 nurses in their third year not one failed to come up to the standard required for a certificate; of 12 in the second year not one failed to obtain over 60 per cent. of the marks; and with regard to the 11 first-year nurses, all showed evidence of very careful teaching and a very reasonable standard of general education, which the examiner said had not always been the case in past years.

### THE REPORT OF A MEDICAL INSPECTOR.

In a report to the Local Government Board on the nursing arrangements in the sick wards of Bedford Workhouse, Dr. Fuller, medical inspector, admits a great improvement since his previous inspection, but says that the staff is still inadequate. He says that a general principle in nursing in workhouses, specially applicable at Bedford, is the fact now generally accepted by Guardians, that helpless or wet and dirty cases, or cases bordering upon or actually suffering from senile dementia, require much more skilled care and attention from suitably trained nurses than average cases of illness, such as pneumonia, or rheumatic fever, after the very acute stage is passed. Therefore they necessarily take up more of their time both day and night. We are not sure that the fact is so generally accepted as Dr. Fuller implies, but its recognition, whether at Bedford or elsewhere, is not of much value unless it is accompanied by the strengthening of the nursing staff.

### The Nursing Outlook.

"From magnanimity, all fears above;
From nobler recompense, above applause,
Which owes to man's short outlook all its charm."

### THE NEED OF THE TRAINING SCHOOLS.

We have had the privilege, which we greatly appreciate, of reading the confidential reports of the matron of the largest training school in this country. These reports cover a period of thirteen years, and show in full detail with great ability the developments which have taken place and the improvements in the lot of the probationers and nurses which a wise administration has gradually introduced. We propose to bring out the leading facts in connection with the work in question during the next few weeks, because we are confident that they must prove of great assistance and encouragement to all who are engaged in the training of nurses.

The attitude of the matrons of the greater training schools, or a majority of them, in holding aloof from what may be described as the public work of nursing, has often been adversely criticised. The more this question is examined the more we believe will it be found that the matrons in question have had to take their choice between neglecting their duties to their individual hospitals and the staff for whom they are responsible, and devoting considerable time to public matters which directly have little or no bearing upon their official duties. Judged from this standpoint there can be no question that the first duty of every matron or lady superintendent is to her own hospital, and the immediate duties which devolve upon her in connection with her work there. If the individual matrons had taken a different course, the actual developments and improvements, which have made the English schools of nursing an example which other nations have been glad to follow, could not have made the great progress which they have done in fact. When a responsible administrator has to choose between the discharge of all the duties which immediately pertain to her office, and the neglect of those duties in pursuit of public work which might add to her popularity and public reputation, the decision in favour of the former course must prove paramount. All thoughtful and responsible people ought therefore, in the existing conditions of our hospital training schools, to honour the women who have had the courage to stick to their last and to make their schools efficient as a primary duty.

This question when looked at from the point of view of the responsibility of the governing committees who control the training schools assumes, however, a different complexion. As the education of nurses has attained higher and higher standards of proficiency, it has become more and more essential that each great training school should be controlled by a lady principal of wide experience and special knowledge, and that she should be relieved of most of the routine duties which have hitherto proved such a strain upon the energies of the majority of English matrons. We believe that the time is not far distant when every great English hospital will modify its system by appointing a Principal of its training school, who will have under her an adequate number of trained and able assistants, so that she, as the chief administrator and responsible head of the whole organisation, may be able to have sufficient leisure to devote the whole of her energies and mind to supervision, initiation and necessary discipline. If this modification in system is once brought about, the principals of training schools will be given the position which they ought to occupy, not only in connection with the schools but vis-a-vis with public opinion and its proper guidance in nursing affairs. Despite the fact that the old methods of running the nursing and female departments of English hospitals have under modern developments become inadequate, and in some respects out of date, there can be no doubt that, owing to the splendid character of many of the women who occupy the position of matron or lady superintendent in our hospitals, the work carried out under their supervision has exhibited a thoroughness which has made it the admiration of all whose opinion is best worth having. As we have before pointed out, however, this result has sometimes been attained by too great a strain upon the women administrators.

The position of nursing in these islands at the present day necessitates a reconsideration of the older methods, and the sooner the necessary modifications are introduced in the directions we have indicated, the sooner will the completest efficiency be secured and a guarantee afforded that every wise and possible development will certainly be introduced into each great training school. Public opinion in these days requires guidance by those who have thoroughly mastered nursing in all its aspects, and we look to the committees of our great hospitals to take prompt and effective action in this matter. It is very difficult for those who have been accustomed to run a business on certain lines to realise that the time has come for the modernising and improvement of old methods. At present there are certain weaknesses in the methods of the training schools, which ought to be able to command a paramount voice wherever nursing is concerned. This they may certainly obtain by a prompt recognition of the facts which we have endeavoured to bring out in the present article.

### Abdominal Surgery.

By HAROLD BURROWS, M.B., F.R.C.S., Assistant Surgeon to the Seamen's Hospital, Greenwich, and to the Bolingbroke Hospital, Wandsworth Common.

### INTESTINAL OBSTRUCTION.

A FREE passage for the intestinal contents from the stomach to the anus is essential for the well being of every individual. Even if a slight obstruction is present definite and troublesome symptoms are apt to arise; and if the obstruction is more than slight a marked disturbance of the patient's health is certain to result. Before giving an account of the symptoms of intestinal obstruction it may be well to mention some of the more common causes.

### CAUSES.

One of the most frequent of these is fæcal accumulation. In patients who have suffered from habitual constipation the lower bowel may become so loaded and distended with dry, hardened fæcal material

THE STOMACH AND INTESTINES.

1. Duodenum. 2. Jejunum. 3. Ileo-cæcal valve. 4. Appendix. 5. Cæcum. 6. Ascending colon. 7. Hepatic flexure of colon. 8. Splenic flexure of colon. 9. Transverse colon. 10. Descending colon. 11. Sigmoid flexure. 12. Rectum.

that it no longer has the power to evacuate its contents, and so a condition of true intestinal obstruction becomes established.

Occasionally the intestine is blocked by a foreign body which has been swallowed, by a mass of indigestible food, by a large gall-stone which has ulcerated through into the bowel from the gall-bladder, or by a new growth.

In another class of cases the bowel becomes strangulated in a hernial sac or by adhesions within the abdominal cavity. In yet other instances obstruction is due to twisting of a loop of intestine (volvulus), or kinking caused by adhesions. Or there may be narrowing of the bowel at one or more points due to ulceration, malignant or otherwise, of the intestinal wall. Lastly, a portion of bowel may become invaginated into the part immediately below (intussusception) and so bring about obstruction. The principal symptoms are colic and constipation. If the obstruction is complete other signs develop in addition to these, including vomiting, distension of the abdomen, and sometimes visible peristalsis.

### COLIC.

The general features by which intestinal colic may be distinguished from the pain of peritonitis have been explained in a previous article, and it is not necessary to go further into the matter here than to recapitulate the characters of colic. The pain is intermittent, or in severe cases there may be constant pain of a lesser degree with marked exacerbations from time to time; whereas in peritonitis the pain is constant and nearly uniform. In mild cases the abdomen is not tender to pressure, and even in severe cases the tenderness is not so acute as in peritonitis. In simple cases, too, the other characters of peritonitis are absent in the early stages as a rule, although certain acute kinds of obstruction, such as a strangulated hernia, may show at the commencement signs resembling those of peritonitis. But as the diagnosis is a matter that concerns the doctor rather than the nurse, the points of difference and distinction between colic and peritonitis will not be discussed at greater length.

The commonest cause of intestinal colic is constipation, and in this case the pain is relieved when the bowels have acted. To secure the action it is better to give an enema or a glycerine suppository than an aperient by the mouth. The former will produce the required effect more rapidly. Moreover, if the colic chances to be due to some physical obstruction in the intestine, a dose of aperient medicine may seriously aggravate the patient's condition. If the colic is very severe some relief may be obtained by applying hot fomentations or turpentine stupes to the abdomen. In applying a turpentine stupe care must be taken that the turpentine is evenly distributed on the flannel, or whatever material is used, because a quantity of turpentine applied to one spot on the skin may cause it to become blistered and sore. If then it becomes necessary to open the abdomen, a sore and blistered skin greatly adds to the dangers of the operation, as it is impossible to disinfect the skin properly when it is in this condition. But the mistake is often made, and is due to the common method of wringing a piece of flannel out of hot water and then sprinkling the turpentine on to the flannel and immediately applying this to the abdomen. The correct way of using the turpentine is to sprinkle it on the flannel before the latter is wrung out. This method ensures an even distribution of the turpentine so that blistering is less likely to result.

Simple fomentations which are too hot when applied, and also too vigorous efforts to disinfect the skin, are other ways in which this troublesome complication of abdominal surgery is produced.

In some instances intestinal colic is due to spasm of the bowel; when this is the case, belladonna or opium, given by the mouth, may bring relief.

If intestinal colic frequently recurs there is always need of suspecting the presence either of partial organic obstruction—such as might be due to adhesions or new growth, or of some constitutional affection, especially lead-poisoning and locomotor ataxia.

### Constipation and Vomiting.

In severe intestinal obstruction, as occurs in strangulated hernia, there is complete constipation, and neither fæces nor flatus are passed. It is a curious thing, but this usually is true even if the obstructed point is high up in the small intestine. The symptom is a very important one, and great reliance may be placed upon it as proving the presence of obstruction in cases which are otherwise doubtful. Vomiting due to obstruction is incessant —provided the patient has not been given opium or morphia—and to some extent characteristic. At first the contents of the stomach are evacuated, then the vomit becomes bilious, then brownish, and lastly it comes to have a fæcal odour. This alteration in the character of the vomit is an important aid to diagnosis, and therefore samples should be preserved for the surgeon's inspection.

The symptoms of intestinal obstruction, including the colic and vomiting, sometimes may be quite arrested by a dose of opium or morphia. Therefore, these drugs should be withheld until the diagnosis is decided; because, in the absence of the chief symptoms, intestinal obstruction is likely to be overlooked and operation delayed, with disastrous consequences. For with intestinal obstruction, as with acute peritonitis, the patient's hope of recovery lies in early operation.

Once the diagnosis has been made and operative measures decided upon, opium may be given with the greatest benefit to the sufferer.

### Course.

The course of a case of intestinal obstruction will depend largely on the nature of the lesion, and upon its situation.

In partial obstruction, such as occurs in the course of malignant disease of the large intestine, there are often spells of severe constipation alternating with attacks of diarrhœa, and this sequence is rather characteristic of partial obstruction low down in the bowel. With partial obstruction high up in the small intestine, colicky pains, vomiting, and loss of weight are the salient features.

The course which an unrelieved case of complete intestinal obstruction runs is dependent largely upon the situation of the lesion. Thus, obstruction of the large intestine is usually chronic, that is to say, in untreated cases the patient may live two or three weeks after the obstruction has become complete; whereas in complete occlusion of the small intestine death will ensue in a few days unless the patient is relieved by operation.

The treatment of almost all cases of genuine intestinal obstruction is operative. But there are other therapeutic measures which are needed in addition.

When a patient with supposed intestinal obstruction has been admitted to hospital, the first thing to do after he has been settled in bed is to administer an enema. If the case is one of true, complete intestinal obstruction, it is nearly certain that neither flatus nor fæcal material will be brought away by the injection.

Hot fomentations may be applied to the abdomen to relieve his pain, and if he is at all faint and cold or suffering from shock, hot bottles will be needed.

For the vomiting, the best thing to do is to wash out the stomach, a procedure which will have to be repeated, probably, after the operation is over and before the patient is removed from the operation table.

So soon as the diagnosis has been made, opium or morphia may be given and will bring the patient the greatest relief; these drugs will stop his pain and vomiting, quiet his heart, and greatly improve his general condition.

But these measures are all subsidiary to the early operation, which is the only means we have of preventing death in cases of intestinal obstruction.

### The Nurses' Clinic.

### THE DISTRICT NURSE AND SCARLATINA.

Scarlatina latens, or latent scarlet fever, is very common and peculiarly dangerous to persons other than the patient. He himself suffers comparatively little, but he is nevertheless capable of passing on the infection in a severe form. He has usually what passes for a slight cold and sore throat, but later on there is an appearance as of dropsy and a general desquamation or peeling of the skin. All children known or believed to have been exposed to infection should be closely observed, care being taken not to alarm them, and at the first distinct sign of ailing they should be placed in quarantine until the matter decides itself.

It must not be forgotten that many mothers have quite as much practical experience with regard to scarlatina, measles, etc., as the nurse trained in a general hospital, and in slight cases a considerable minority of them deliberately conceal symptoms with the import of which they are well acquainted. The suspicion with which all careful mothers look on a neighbour's child who has been kept at home ten days or a fortnight on some specious pretext, is often too well founded. The nurse must try and raise the standard of honour and unselfishness in these matters, and can most effectually do it by pointing out the desolation that may be spread by a single one of these wilfully concealed cases.

Scarlatina simplex is the ordinary form of scarlet fever. It begins with a sense of discomfort in the throat, gradually increasing to pain, with considerable difficulty in swallowing, there may be vomiting and high temperature, and there will probably be headache, shivering, weakness, and lassitude. The characteristic eruption generally appears on the second day on the neck, chest, forearms, abdomen, and thighs, completely covering the body in about twenty-four hours, and remaining at a high point of development for one to three or four days. The rash at first consists of minute pink papules; these increase in size and redness and presently join together, so that the skin is completely covered with a flush, varying from a light red that may

THE NURSES' CLINIC—*Continued.*

escape detection to a deep crimson. Small bladders (sudamina, the result of sweating) may occasionally be seen containing a pale yellow fluid. The face, hands, and feet are sometimes swollen; the tongue is furred on the centre and sides, but red at tip and edges; the tonsils are greatly enlarged. Between the fourth and sixth day the temperature falls, the eruption fades, the patient begins to feel much better, and peeling of the skin sets in. The period of desquamation varies greatly; in some cases it is completed by the end of the fourth week, in others it may be eight, and in rare instances as much as twenty weeks. It is not definitely known whether infection can be conveyed by the scales, but it is a wise precaution to keep the patient well greased with carbolised oil, 1-40, and give him a warm bath at least once daily; to assist in getting the scales completely off the feet they may be well soaked in hot water, care being taken to avoid chills. The mouth and throat should be frequently gargled with boracic or Condy's fluid. It must never be forgotten that the germs of the disease have extraordinary vitality; many cases have been traced to clothing, playthings, etc., infected several years previously.

About the third week the patient not seldom takes "a turn for the worse," temperature rises, appetite is lost, and kidney disease declares itself. It is well to bear in mind that nephritis frequently follows mild—and perhaps, therefore, carelessly treated—cases of scarlatina, and may be of a severe and even dangerous character.

In Scarlatina ulcerosa one or both tonsils become ulcerated, the glands of the neck may swell, and serious abscesses form; the nose and ears may also be affected. Inflammation of the tissues of the neck sometimes follows, and may reach as far as the breast bone and the outer ends of the two collar bones. These complications add considerably to the length of the illness, and to the possibility of lasting ill effects on hearing and eyesight. Other complications frequently occurring are laryngitis, rheumatism, pneumonia, and convulsions.

In Scarlatina maligna the eruption is dark, there is extreme prostration and feebleness of pulse, and death quickly results.

Scarlatina is most common in autumn and winter; it is found all over the world, but especially in temperate climates. The period of incubation may be as little as three hours, and is rarely as much as seven days. One attack usually protects from another, but not invariably.

The treatment depends partly upon the severity of the case and the age and constitution of the patient, but the sufferer must always be confined to one room and an even temperature for at least four weeks, and must be isolated until desquamation has definitely ceased. While seriously ill the patient should have beef-tea, chicken broth, milk, eggs, and bread. A tepid bath should be given once or twice daily; the mouth and throat must be kept clean, the nose and ears syringed, and the eyes bathed when necessary. If the glands of the neck are swollen they should be protected by cotton-wool. When the temperature falls the patient must be given a flannel shirt, and the room should be kept at 60° F.

All mothers of young children should be warned that the majority of cases of scarlet fever occur before five years of age, and that the most fatal period is between three and four. They must be encouraged to believe that if they can protect their children during that time they will probably escape the disease altogether, and if they do contract it they will almost certainly have it in a lighter form and have more strength to resist the attack. For this reason, if for no other, all mothers who have a decent home and time to attend to their children, should keep them away from school up to the extreme legal limit, and if they are at all delicate should apply for special exemption. At school they will almost inevitably be brought into contact with many uncared-for little creatures, some of whom may well happen to be in an infectious stage of scarlatina, measles, mumps, or diphtheria.

### Incidents in a Nurse's Life.

A GUY'S NURSE AT CAWNPORE.

Night-duty in India, which is so often trying in England, is delightful in Cawnpore. The air is soft and fresh and full of new sounds. All through the hours the lizards sing, such bright-eyed, pretty, yellow-green creatures, who sit on the wall and catch moths, and then when morning comes go away and hide. The moths are as big as birds, the ants, crowds of them, as big as beetles, whilst every now and then a fat frog hops into the hospital to have a look round. The stars are perfectly wonderful, almost like lights in a canopy of darkness, and the heat is so great that the patient cannot be covered; legs in pyjamas, and various coloured jackets are visible everywhere. The native women always wear skirts in bed and the hospital provides them. By the sides of the beds lie various sleeping figures, the relatives of the patients who stay near to see that no harm is done to them and that their food is not polluted by the nurses touching the vessels containing it. In the early morning the crows, minas, weaver birds, and pigeons set up a joyful chorus. Then work with out-patients soon commences. The wounds are a nightmare, they are so huge and apparently hopeless. After the doctors have finished with plague for the season—for it always comes with the cold weather and goes away with the heat—there are always numerous patients with plague abscesses coming up every day to be dressed. Part of the treatment is to open the abscesses as soon as they are ripe, and they are always a long time in healing up. Ever so many are in the groin, and they have to be dressed while the patient squats on the ground and the nurse sits on her heels! The native nurses are most untrustworthy and need buckets of patience flowing in a perpetual stream. The same thing has to be told them every day, and even then the English sister has to see that it is done. They are more particular than English nurses about not doing other people's work, or anything the servants ought to do. "To-morrow will do," is their attitude towards everything.

Monkey-worshippers are rather a trial. One of them asked the lady doctor if "it was written in the book of our God that she would get well." On being told that it was not written she said it would be in the book of the monkey god, and she insisted upon going home. Another monkey worshipper came to the hospital with her baby, and whenever the baby was taken out of its cot she put a monkey's skull in its place, because, she said, "it is our custom."

### To Nurses.

We invite contributions from any of our readers, and shall be glad to pay for "Notes on News from the Nursing World," "Incidents in a Nurse's Life," or for articles describing nursing experiences at home or abroad dealing with any nursing question from an original point of view, according to length. The minimum payment is 5s. Contributions on topical subjects are specially welcome. Notices of appointments, letters, entertainments, presentations, and deaths are not paid for, but we are always glad to receive them. All rejected manuscripts are returned in due course, and all payments for manuscripts used are made as early as possible after the beginning of each quarter.

### Women and Thrift.

On the afternoon and again in the evening of Monday, May 28, a representative audience of ladies gathered in the Council Chamber of Denison House, Vauxhall Bridge Road, to discuss what women had done and what women could do in the matter of saving. The afternoon discussion was opened by Mrs. Alfred Pollard, of the Women's Institute, who dwelt upon the difficulties which women experience in saving. Of these difficulties the first is that women, having for countless generations been in the habit of depending on man for maintenance, had no hereditary disposition to thrift. The second, and at the present day probably the more important is that woman's earnings are so small that it is almost impossible for them to save, or at least their savings are so trifling that at the utmost reckoning they cannot put by enough during their working years to keep them comfortably in old age. Mrs. Pollard admitted that by a system of stinting, if not starving, of body and soul a woman might save more than the £10 a year which she had set down as the reasonable saving possible to a professional woman out of an income of £150—which sum Mrs. Pollard regards as the average earnings of such a woman as she was speaking of. She pointed out that women were only too prone to such small economies, the wisdom of which was more than doubtful, and that they wanted rather to be taught to spend money wisely than to save it foolishly, and she added "looking round me at the inducements offered to them to save it wisely, I see so few that I must own that I have never yet had the courage to address any body of educated working women on the duty of thrift at all."

It is doubtful if Mrs. Pollard received much encouragement from those who came after her. Mrs. Loch, Secretary of the Order of United Sisters, told of the methods of that Society, especially of one of its courts—that named "Work and Leisure"—which is meant to appeal especially to women who feel the need of larger sums than the women of the industrial class, who form the bulk of the members of the Order. The members of Court "Work and Leisure" can obtain sick pay ranging from 8s. to £1 a week, a funeral allowance of from £6 to £12, and a pension of 10s. a week after the age of sixty-five. After Mrs. Loch came Mr. Alfred Watson, Fellow of the Institute of Actuaries, who gave a great deal of statistical information regarding the sickness risk in friendly societies for women. Afterwards, when the meeting was thrown open to discussion, an amusing dispute arose between Mrs. Pollard and some of the other speakers on this subject of sickness risk, and the cost of it to friendly societies. Mrs. Pollard contended that women of the better classes made fewer claims for sick pay than the average working woman, because they were less willing to give in, had more courage, and a higher sense of duty. But this contention was not supported by Mrs. Loch, whose experience in the United Sisters did not confirm it, while Miss Page of Norwich, who represented the Women Foresters, said that the professional women in their society were by no means less exigent in the matter of sick pay than others. And Mr. Watson also gave no encouragement to the idea that gentlewomen either suffered less than their humbler sisters from the ills that flesh is heir to, or bore up more bravely under them.

In the evening meeting, the first item was a brilliantly unconventional paper by Miss Clara Collet. She protested, as Mrs. Pollard had done in the afternoon, against women workers, whose earnings were but small, making their present lives more wretched and cramped by putting by enough "to pass a half-starved old age without coming on the rates." "In their place," said Miss Collet, "I would far rather make the most of my life now, and in old age devote myself to making life in my workhouse ward a little more breezy and interesting." This philosophy she emphasised in her concluding axioms: "The object of saving should be spending"; and "Saving which checks growth is uneconomical." Miss Collet was, however, far from discouraging mutual help, but advised that the appeal for joining any benefit society should be founded rather on sympathy and altruism than on selfish fear for the future—fears which do not appal the young, and which the old realise only when it is too late. This point was subsequently dwelt on by several speakers, who pointed out that the terroristic method of advocating friendly societies was likely to draw into their ranks the "bad lives" which were sure to be a burden upon the funds, and would not appeal, as the higher motives might do, to the healthy and vigorous, those not likely to make frequent demands for sick pay, who must be the backbone of any mutual society.

Miss Page, who followed Miss Collet, gave some interesting details of the work of friendly societies, especially in the matter of sick pay. Her illustrations were largely drawn from the experience of the Foresters' Society, to which she belongs, and offered convincing proof of the value of such societies to women whose earnings are so small as to make it almost impossible for them to save individually for the emergencies of life. Finally, Miss Poole read a short paper in which she advocated the National Deposit Friendly Society, which Miss Page had spoken of as in many ways unsatisfactory, as the best method of saving for domestic servants. Some other points were touched on in the subsequent discussion, as, for example, the possibility of an allowance to members when they are out of work, and of a surrender value for contributions which members could claim if at any time they wished to withdraw from their society. But the general impression seemed to be that however desirable such additional benefits might be, to give them would involve the demanding of such high contributions (comparatively speaking) that working women could not afford to pay them. The whole question thus seemed to hark back again to the matter of wages, and the moral to be that women cannot save enough because they do not earn enough. That women have no innate incapacity for saving was shown by Mrs. Sunderland, who had acted as a travelling matron to parties of servant girls going out to South Africa, and who had found that of those with whom she had kept in touch, a large number were saving considerable sums out of the good wages they could earn there. It seemed strange that in a conference dealing with thrift for women, no mention should have been made of the existing societies for enabling professional women to save, such as the Royal National Pension Fund for Nurses. Also it seemed strange that no one brought forward the fact, which must be well known to those who have to do with women workers, that a pension which begins at sixty-five is quite unattainable to a woman without private means, even though she might live to enjoy it. A woman's working life does not extend to sixty-five. Formally or informally, she is compulsorily retired long before that, and if a pension is to help her it must begin at least ten years sooner. But as it was resolved to appoint a committee of seven to investigate the whole matter, these points will doubtless be dealt with in due course.

### "The Hospital" Convalescent Fund.

The Hon. Secretary begs to acknowledge with thanks the receipt of 7s. 6d. from the Travel Correspondent.

### The Royal National Pension Fund for Nurses.

The nineteenth annual general meeting of the Royal National Pension Fund for Nurses was held on Wednesday afternoon at River Plate House, Finsbury Circus. The adoption of the report was moved by the Chairman, Mr. Everard A. Hambro, seconded by Sir Henry Burdett, and supported by Miss Swift, matron of Guy's Hospital, and Mr. Pollitt, Treasurer of the Blackburn District Nursing Association.

### REPORT OF THE COUNCIL.

The Council have pleasure in submitting the report with the annexed accounts and balance-sheet for the year ended December 31, 1905.

### Pension Branch.

The number of policies issued during the year was 1,304, assuring immediate annuities of £684 1s. 7d., and deferred annuities of £20,440 12s., and producing in annual premiums £12,822 19s., and in single premiums £26,955 10s. 8d. This shows a considerable increase over any previous year. The total premiums received during the year amounted to £105,468 3s. 9d., being an increase of £13,592 7s. 5d. over 1904.

*Withdrawals.*—The number of policies surrendered during the year was 419; these were held by 396 nurses. The average number of policies discontinued becomes steadily less.

*Pensions Falling Due.*—Eighty-three nurses became annuitants during the year; the total number of nurses drawing annuities on December 31 was 627, receiving at the rate of over £14,000 a year, the average annuity being over £21. Each year a proportionately increasing number of annuitants take out fresh policies.

### Sickness Assurance Branch.

One hundred and thirty-three policies were issued during the year, assuring weekly sick pay of £94, and producing in annual premiums £267 2s. The premiums received during the year amounted to £1,693 19s. 5d., and the amount distributed to 259 nurses amounted to £1,786 17s. 7d.

### Invested Funds.

The total invested funds at the close of the year amounted to £994,571 14s. 7d., being an increase of £94,527 9s. 6d. over 1904.

### Reserve Fund.

The usual principle of crediting all interest over 3½ per cent. to this fund has been followed. The Reserve Fund now stands at £29,292 8s. 10d.

### Expenses of Management.

The expenses of management amounted to £4,535 8s. 9d., being at the rate of under 4½ per cent. on the premiums received.

### Junius S. Morgan Benevolent Fund.

The usual report of this fund is presented. The Secretary's salary has again been provided by generous donors, who wish to remain anonymous. To the President (Lady Rothschild), the Hon. Secretary (Mrs. Pritchard Binnie), the Secretary and the members of the Advisory Committee the Council are much indebted.

### Council.

The members of the Council retiring by rotation are: Sir Henry Burdett, K.C.B., Mr. Edward Rawlings, Mr. Arthur H. A. Morton, Mr. Walter S. M. Burns, and Mr. C. Eric Hambro, M.P., and, being eligible, offer themselves for re-election.

### Representatives of Policy-holders.

The Articles of Association provide for the election of representatives of the annuitants and policy-holders of the Society as members of Council. Miss Fisher has resigned, and for her services the Council have accorded to her their cordial thanks. The two ladies retiring by rotation are Miss Mabel Cave, matron, Westminster Hospital, and Miss P. Peter, late general superintendent, Q.V.J.I.

The following two ladies have been nominated in proper form, namely—Miss Macfarlane, matron, Victoria Infirmary, Glasgow, and Miss Chambers, matron, Ancoats Hospital, Manchester, and the Council have submitted these four names to the policy-holders to fill the three existing vacancies.

### Auditors.

Messrs. Whinney, Smith, and Whinney, chartered accountants, retire in accordance with the Articles of Association, and, being eligible, offer themselves for re-election.

By order of the Council,

Louis H. M. Dick, Secretary.

---

### THE JUNIUS S. MORGAN BENEVOLENT FUND.

During the past year more applications have been made than during any previous year, with the exception of 1902; the expenditure in grants, etc., accordingly shows an increase on that of 1904.

There have been 105 applications for assistance, in several instances from nurses who have been previously assisted by the fund. Forty-seven nurses may be considered as being permanent annuitants in receipt of weekly or monthly grants. During the year the deaths of four annuitants occurred, and their places were filled by fresh applicants.

Twenty-nine nurses received grants during illness, either to enable them to obtain rest and change of air at convalescent homes, or extra nourishment, medicine, etc.

The premiums of seven nurses were paid, and for three nurses the necessary artificial teeth and spectacles were obtained. Substantial grants were made in six cases to assist in paying debts, etc., occasioned by illness. One nurse, 65 years of age, was granted a donation to enable her to accept the offer of a comfortable home in British Columbia, where her previous nursing experience would certainly enable her to find employment. Other nurses were helped in various ways, including payment of rent, increase of grants, etc.

### The Usefulness of the Fund.

The following letters illustrate the usefulness of the fund, and show a keen appreciation of it on the part of the nurses. Nurse E., who had been in receipt of constant help during a long period, writes :—

"I am very glad to tell you that I have started work again (June 1905) after undergoing open-air treatment for four years. Through the kindness of the Committee in granting me assistance from the Benevolent Fund during a long period of that time, I have been enabled to continue the cure longer than I could otherwise have done, and I express my deepest gratitude to the Committee for the help received from them."

Nurse A. R. writes :—

"I wish to render my sincere thanks to the Committee for their great kindness in helping me all this time. The weekly grant has been a great help to me in meeting extra expenses, also in helping me with my monthly premiums. I feel that the Royal National Pension Fund is a grand investment, and I shall do my utmost to persuade others to become members."

The usual Christmas gifts of 5s. were made to 36 of the oldest annuitants, and warm knitted garments, sent by kind sympathisers, were much appreciated by the recipients, some of whom are confirmed invalids.

The donations and subscriptions exceed by a few pounds those of 1904. On the other hand, grants, etc., paid out have been greater than in any previous year, with the exception of 1902, when subscriptions and donations amounted to £2,060. Having regard to this increase of expenditure, a special appeal is made to the nurses to contribute, one and all, 1s. a year, and to further show their interest and co-operation by continuing to interest their friends in the Benevolent Fund.

The Committee desire to thank all those who have contributed to the fund, and also to express their warm appreciation of the valuable services and assistance rendered by Mr. Dick.

E. L. Rothschild, President.

M. B. Farmer, Secretary.

### Asylum Workers and Long Hours.

Sir James Crichton-Browne presided at the annual general meeting of the Asylum Workers' Association last Friday, in place of Sir John Batty Tuke, M.P., the President. In moving the adoption of the report, Sir James said that, though the number of members of the Association was 3,277, it was not as large as it ought to be, for it was calculated in the report that there were 20,000 persons engaged in the care of the insane in the asylums of the United Kingdom. Their numbers ought to be brought up to eight or nine thousand. The Select Committee of the House of Commons which had been considering the question of the State Registration of Nurses had, with regard to the registration of asylum nurses and attendants, adopted almost the exact words of a memorandum he had submitted to them, and had recommended that a separate register of mental registered asylum nurses should be kept by the central body of those nurses who had served for not less than three years and had received the certificate of the Medico-Psychological Association, and could produce satisfactory evidence of good character. He was certain that this Association would have a seat allotted to it on the Central Board. He felt confident that the nurses and attendants of this Association would not wish in any way to separate themselves from the medical men under whom they had to work and to whose kindness the success of the Association was largely due. All the best nurses would be opposed to any such registration in which the power would be allowed to lapse into the hands of self-assertive women, when the whole thing would become a farce and a failure. It would be a great assistance if the Association would pass a resolution in favour of an equal representation of nurses and medical men on any board. Their modest and legitimate demands for pensions had not yet been satisfied, but he was in hopes that they might be before long. The question of lunacy was a by-path in public affairs, but it could not be ignored much longer, as there were now 120,000 certified lunatics, and the number was increasing in much larger ratio than the general population.

Dr. Hyslop seconded the motion; and Dr. Cooper, M.P., speaking in support of it, as a late member of the Asylums Committee of the London County Council, maintained that fourteen hours a day was too long for asylum attendants to work. Eight hours was quite enough, considering the exhausting, anxious nature of their work. He did not see why the Association should subscribe a penny to support homes of rest; these it was the duty of the public to supply.

Mr. Will Crooks, M.P., who also supported the motion, considered that there was no service given to common humanity that could compare to the service given to the insane. If anyone wanted to know the depth of human love he should go into an asylum and see the care and attention afforded to the lunatics. No woman ought to be allowed to devote more than eight hours out of twenty-four to such trying work. There should be more attendants. The ratepayer ought not to count in a case like this.

An alteration in the bye-laws of the Association approved by the Executive Committee having been passed, the gold and silver medals for long and meritorious nursing service were presented. The awards were as follows : Gold medals —1, Attendant P. Bolton, Moorcroft House, 45 years 7 months' service; 2, Nurse E. Cooke, late Colney Hatch, 35 years 11 months. Silver medals—1, Attendant G. Collins, Colney Hatch, 34 years 2 months; 2, Nurse H. J. Robinson, Middlesex C.A., 29 years 7 months.

### Where to Go.

New Hospital for Women, 144 Euston Road, N.W.—Thursday, June 14, 2.30 to 7 p.m. Sale of children's clothing, toys, dolls, books, and other articles.

### The Royal British Nurses' Association.

The annual meeting of the Royal British Nurses' Association was held at the Imperial Institute on Thursday last, Sir James Crichton-Browne presiding. He was supported by Dr. Comyns Berkeley (Medical Hon. Secretary), Dr. Clement Godson (Hon. Treasurer of the General Funds), and Mr. John Langton (Treasurer of the Settlement Fund).

### The Financial Position.

The preliminary business of reading and confirming the minutes of the previous meeting, the appointment of scrutineers, the reports of the Hon. Treasurers and the Executive Committee, with the election of the General Council was soon despatched. The reports showed that the financial side of the Association can hardly be regarded as in a satisfactory condition, owing to the resignation of members, unpaid subscriptions, and sundry unexpected expenses. The *Nurses' Journal* accounts, too, showed a deficit of £20 13s., instead of a surplus of £9 8s. 11d., which was the result in the previous year. The balance in hand at the commencement of this year was £11 17s. 6d., as against £112 3s. 2d. at the beginning of 1905, showing a loss under this heading of £100 5s. 8d. The printing account was somewhat less than formerly, but in the autumn of the present year the Triennial Roll of Members would have to be published, at a cost of little less than £100. The Settlement Home at 20 Clapton Square was not as flourishing as could be wished, though a revision of the rules had set it upon a better basis and increased its attractiveness, with the result that several applications for admission had been made, and there were now six nurses resident in the Settlement. On the other hand, the Helena Benevolent Fund, which makes grants to members in times of sickness or difficulty, presented a satisfactory report. During the year benevolent grants to the amount of £60 had been made, as against a total of £40 18s. in the previous year; and the invested stock of the Fund remained at the sum of £1,372 6s. 6d., giving an interest of £37 3s.

### Votes of Implied Censure.

Strictly official business being concluded, the meeting turned its attention to the burning question of the day, and Miss Forrest rose to move the first of three resolutions standing in her name on the agenda—namely, "That the Bill for State Registration of Nurses as drafted and presented by the Executive Committee of the Royal British Nurses' Association does not express the feeling of the majority of the members of the Association, as, in their opinion, it does not provide for adequate representation of the nursing profession in the constitution of the Central Board." Miss Forrest claimed that her views were those of a large number of nurses, including such well-known authorities as Miss Cureton, Miss Sidney Browne, and Miss Jones. She emphasised the importance of unity in the matter, and appealed to the officers and executive to remove a stumbling-block in the way of securing unanimity. Miss Edla Wortabet seconded the resolution, and referring to the progress of the profession and its improved status, declared that modern nurses, being no longer uneducated as a body, were fully capable of forming a self-governing institution.

Miss Clarke, who avowed herself an enthusiastic supporter of the resolution, said that they were responsible not only to the small body of women represented in this room, but to the 80,000 nurses working all over the kingdom. She vigorously attacked the idea of medical control, and used terms of unmeasured reproach directed against those who had drafted the Bill.

Miss Gordon followed with a strong defence of the rights of medical men to be fully represented on the Central Board. She thought that it was a pity the question of woman *versus* man should come into such a matter at all, and she trusted that they were not there to represent the "new woman." Mrs. Latter warmly supported these views, and Dr. Comyns Berkeley pointed out that the statement about the 80,000 nurses was quite incorrect—that number would include nursemaids of thirteen to seventy.

The resolution was lost by a large majority, as were also the other two moved by Miss Forrest—namely, "That in the interests of the nursing profession there should be a fixed majority of nurses on the Central Board elected from amongst the nurses to be placed on the State Register," and "That the members of the Association assembled in general meeting hereby protest against the unbusinesslike and unjust conduct of the honorary officers of the R.B.N.A. at the meeting of the General Council held on February 7, 1906, and now place on record their disapproval of the procedure adopted at such meeting."

Sir James Crichton-Browne and "Autocratic Matrons."

The last resolution provoked an animated discussion as to the exact nature of the charges of "unbusinesslike and unjust conduct," and Miss Clarke complained bitterly of the impoliteness of the Medical Secretary in replying to her assertion that she had not received the voting-paper.

In summing up Sir James Crichton-Browne explained that the Bill had been passed in the usual way at the general meeting, and that their decision must be taken as authoritative. He wished to remind the nurses that in the matter of registration the medical profession had no axes of their own to grind, their attempts to further it were purely in the interests of their patients, to secure for them efficient and faithful nursing. He did not understand that the Registration of Nurses was proposed solely in the interests of the nurses themselves, though they would, of course, reap great collateral benefits. Surely it was primarily in the interest of the sick and suffering, and the main object of the Bill must be held steadily in view by all. He asked the nurses whether they wished to call into existence a trades union, under the dictates of a little coterie of autocratic matrons, and stump-orators in petticoats, or whether they would have a thoroughly representative Board. It was fully recognised that in the sick-room nurses must act in co-operation with but in subordination to the doctors, and it was in their own interests that their work should not be judged by their sisters alone, but also by the medical profession. He deprecated the vote of censure against the honorary officials as an ungracious and unjustifiable attack, and warned them to proceed cautiously, or the nurse-demagogue might not become unknown.

The meeting closed with a hearty vote of thanks to Sir James and the other honorary officers, whose patience and courtesy were cordially acknowledged.

The Association afterwards met for tea and sociable intercourse at Earl's Court, where they drifted away in groups to enjoy the sights of the Austrian Exhibition.

### The Middlesex Hospital Nursing and Domestic Reorganisation.

Proposals relating to the rearrangement of the nursing and domestic branches of the Middlesex Hospital have received the approval of the Board, and a scheme, already inaugurated, will provide in future for the supply of trained hospital nurses for private cases on remodelled and more advantageous lines. The new system involves the gradual extinction of the Trained Nurses' Institute as at present constituted, for instead of withdrawing nurses from the nursing staff and reserving them entirely for private work, as heretofore, suitable nurses, who have completed three or more years' training, and shown peculiar fitness for work of this nature, will be so employed, and return to their duties in the wards in the intervals between their cases. At present the scheme is merely in the transition stage, and details in connection with its final regulation and management will be specially adapted to meet such particular requirements as may disclose themselves. The control of the department will ultimately be centred in the Lady Superintendent, who will be aided in her duties by the assistant matron and a home sister, and the office of "sister in charge" will then be discontinued. It is confidently anticipated that by this change more efficient nursing will be available to the public, that the nurses themselves will, through repeated contact with the work of the wards, be kept better acquainted with modern methods, and that an appreciable saving will be effected in the cost of the management.

### Appointments.

Baldovan Asylum for Imbecile Children.—Miss A. O. Henry has been appointed matron. She was trained at the Glasgow Sick Children Hospital and the Dundee Royal Infirmary, where she has since been in charge of the children's ward.

Clun Cottage Hospital, Shropshire.—Miss H. E. Arkle has been appointed nurse matron. She was trained at Charing Cross Hospital, and has since been sister at the Military Hospital, Gosport.

Colonial Hospital, Trinidad.—Miss Stella C. Rust has been appointed first assistant to the matron. She was trained at the Taunton and Somerset Hospital. She has since been engaged in private nursing, and was also for a year at the London Hospital.

Cottage Hospital, Gerrard's Cross, Bucks.—Miss F. M. Hobbs has been appointed nurse matron. She was trained at the Royal Sea-bathing Hospital, Margate. She has since been staff nurse at the County Hospital, Dorchester; staff nurse and temporary matron at Ilkeston Accident Hospital; and staff nurse at the Accident Hospital, Mansfield, Notts.

Medical Mission Hospital, Plaistow.—Miss Annie Nixon has been appointed staff nurse. She was trained at Barry Accident Hospital and at the Branch Hospital for Seamen, Albert Dock, London. She has since been staff nurse at the Maternity Charity, Plaistow, and nurse under the Colonial Nursing Association.

Mercer's Hospital, Dublin.—Miss M. Stanley has been appointed sister. She was trained at the Birmingham Poor-law Infirmary, and has since been staff nurse at the Royal Victoria Hospital, Belfast.

Moorfields Eye Hospital, London.—Miss Florence Emily Augusta Lewis has been appointed staff nurse. She was trained at Southport Infirmary, and has since been working at a private hospital in Cardiff.

Royal Hospital for Sick Children, Edinburgh.—Miss Ellice Rènant has been appointed assistant matron, and Miss Grace Hale night superintendent. They were both trained at St. Bartholomew's Hospital, London. Miss Rènant has been night superintendent at the Royal Hospital for Sick Children, and Miss Hale sister. The latter possesses the certificate of the Central Midwives Board.

Union Infirmary, Haslingden.—Miss S. Taylor has been appointed superintendent nurse. She was trained at the Chorlton Hospital, West Didsbury, near Manchester, and has since been sister at Burnley Union Infirmary.

York County Hospital.—Miss Emily P. Tute has been appointed lady superintendent of nurses. She was trained at the York County Hospital, and has since been ward sister, night superintendent, and assistant matron.

### A Book and its Story.

### BENEDICK AT HOME.

Mr. Halliwell Sutcliffe's new book is written in a lighter vein than some previous ones. There is a pleasantly humorous tone about it, and a fresh-air atmosphere which is mentally invigorating. It forms a natural medium for the pen-portraits of the rustic figures, gentle and simple, that move with vivacity through its pages. The particular corner of arcady utilised by the author for his reflections on men and matters is one well known to him as a Yorkshireman. Benedick and his girl wife have been married six months when he begins his book. Apparently they are so perfectly contented that the reader is not treated to many comments on this important fact. His bride, the daughter of a neighbouring squire, he has known from childhood. And a very sweet and wise personality is suggested when he touches upon the subject. For instance, in the following passage, delicately descriptive, we realise this: "Cathy looks once at me, a glance fleeting as our Northern summer, but full of that elemental strength which makes for deep gladness or deep sorrow."

Benedick's and Cathy's home is "a haunt of ancient" peace—an old mansion with historic memories. It has, among other claims, to support these, "a priest's hiding chamber" looking out across the valley to the moor. This now forms a portion of their drawing-room. "The chamber now is furnished cosily; but half the inner wall is left a jagged edge of masonry to lead one back to history; undoubtedly there are ghosts, trustful, faithful ghosts, about this room in which I sit to-night, after mowing many swathes with Tom Lad." Among many musings over the bygone times, with its changes of monarchy, Benedick's thoughts turn to the days of the Lavender ladies. A charming old-world grace hangs around their memories aptly revived by the author. "I came here to-night, here to the hiding chamber, where ghosts congregate, with no thought in my mind save that of the Lavender ladies. These ladies held the true faith to the last, as the Royalists did—the true faith, I mean, which counts gardens worthier than streets, the starlight more tempting than the gleam of crude and minted gold. They are at home here in the priest's hiding-place, for they are friendly with the spirit of the Stuart days. . . Then, too, my fancy pictures them as always in a garden, filled with the gracious, olden blooms of gillyflower, and pink, and auriculas, and double stock. There are bee-hives under the southern wall and rows of ordered raspberry canes, and a dovecote mounting high in the summer blue."

Benedick has an amiable desire, natural to one so happily placed, to hep all and sundry who ask for assistance. His wife and his old man-servant realise that his pensioners are frequently the reverse of deserving. Some of the most amusing scenes are those connected with his philanthropic mistakes. Tom Lad, his man and general factotum, had, with his wife Stylesey, taken charge of Benedick's domain before his marriage. They figure conspicuously in his domestic *régime* after it. On the evening of the home-coming of their master and his bride they await their arrival. "Stylesey, of course, is at the gate to meet us, and Tom Lad, her patient husband; but they are not the first to welcome us. As soon as we turn the bend of the drive the dogs, who are waiting too, as if they scented strange happenings in the air, espy us, and come galloping, tumbling, racing up the road. . . They have not seen us for six months. How many of our two-legged friends would remember and forgive so speedily? Stylesey's face when we reach the gate is a study in fine lights and shades of feeling. In a moment she has appraised Cathy from head to foot—could tell the details of her wearing gear and whether she is truly happy—and the good woman does not know whether to be vastly jealous or vastly glad. She glances, too, at me—a transitory glance that says plainly, 'Oh, you sir! Lordie, of course you'd be happy.' Tom Lad, on the other hand, has a welcome like the dogs. His weather-moulded face is full of wrinkles, full of smiles that find the furrows out upon his face. He stretches out a hand and takes my own as a playful, ill-trained bear might do. 'Lord help us, 't has been lonesome like without ye, sir,' he says. Stylesey incontinently weeps, and dries her eyes with her apron, and stands off and looks again at Cathy. 'I was crying for Tom's foolishness,' she observes at last. 'Time ye came home, sir, for he's getting out of hand.' Tom Lad glances at me sideways, but says nothing; and, to tell the truth, I am mortally afraid that Stylesey will catch my answering look. . . Stylesey is upset this evening. Her natural tartness and her natural wayward softness seemed to have been summoned forth by our arrival after six months' absence. . . ." Tom Lad and his master have a mutual liking and understanding born of comradeship that arises from community of interests between them. "Perhaps no master ever knows his farm man intimately unless he can mow and swathe beside him, can dig and hoe a furrow with him in perfect comradeship. For myself I see always in Tom Lad's eyes that I can do these things with tolerable credit. Cathy will have none of philosophy to-night. 'Do you use your greenhouse as you used to do, Tom Lad?' she asks with that happy laugh of hers. Tom looks at the hilly moor above us—green with bilberry and uncurling bracken-stems—and his smile is something a fine painter ought to set down on canvas. 'More, oh, ay, a lot more,' he vouchsafes at last. 'I felt lonely like without the master, and the good wife seemed i' the same way, for she nattered, nattered, nattered, till she was fair like a clock ticking all day and all the night through. Oh, ay! the greenhouse thrives. A man must do summat, and make the green shoots thrive, or how's he going to be evens up wi' the good wife?'" Later, when Bendick turns in to the greenhouse as of old to smoke an evening pipe with Tom Lad, he is greeted by him with, "I thought ye'd come, sir! Ye and me, we love a greenhouse. 'Tis so simple-like and so chancy-sweet to come by." Tom Lad takes the opportunity to express his ideas of the comparative advantages of married and single life, with the result that Benedick reflects that "The sense of it all is this—that Tom Lad, whom I reverence and slightly fear, approves of my changed life. And so, to run no risk of spoiling a hard-won approval, I leave him to his plants. Very cool and very warm it is in our North country to-night. The quiet dusk, the quiet scent of my recovered land, are dear to me; the six months' wandering was full of charm, but it is a background only to the home return." Tom Lad makes many shrewd remarks. The following, apropos of luck, which he observes resembles his good wife, is worth quoting. "Luck, try to please her and wait her whimsies and be the bonnie lad in his best bib, and she'll never look your way; but take a good stout ash stick to her—or a bendable, slim switch o' hazel—and straightways she'll want to put your boots on for ye."

"A Benedick in Arcady" will repay perusal.

* "A Benedick in Arcady." By Halliwell Sutcliffe. (John Murray. 6s.)

### Notes and Queries.

**REGULATIONS.**

**The Editor is always willing to answer in this column, without any fee, all reasonable questions, as soon as possible.**

**But the following rules must be carefully observed.**

1. **Every communication must be accompanied by the name and address of the writer.**
2. **The question must always bear upon nursing, directly or indirectly.**

**If an answer is required by letter a fee of half-a-crown must be enclosed with the note containing the inquiry.**

*Verses.*

(129) Can you accept the verses I send, which greatly comforted a patient of mine?—*Affliction.*

The Editor is sorry not to accept owing to lack of space.

*In Need of Work.*

(130) Can you help me to find work? I am 67, and have no employment.—*Nurse V.*

We are truly sorry for you, but it is difficult to find work for you at your age. You might apply to the Church Army, Edgware Road, W.

*A Post as Children's Nurse.*

(131) Can you tell me where to advertise for a post?—*Bristol.*

The *Morning Post* and our own journal would probably secure you a post.

*Training as Children's Nurse.*

(132) Can you tell me where I could train as children's nurse in exchange for a certain period of service?—*Cambridge.*

There are several institutions which train, but we know of none which have the arrangement you suggest; but a modified arrangement is made at St. Lucy's Nursery Home, 28 Upper Tooting Road, S.W.

*Employment for a Simple-minded Woman.*

(133) Can any of your readers help to find a home for a simple-minded woman who can work under supervision? Her friends can pay a very small sum.—*Perplexed.*

We hope that some of our readers may help you.

*A Home Needed.*

(134) Can you help me to find a home for a penniless lady who is deaf? She is willing to assist, and is quite well.—*Bournemouth.*

Perhaps some of our readers can help you.

*Floating Kidney.*

(135) In your issue of May 12 you refer to a lecture of Mr. Watson Cheyne's in which he recommends corsets devised by Gallant as of value in cases of floating kidney. Can you tell me where they can be seen or procured, and give me an idea what they cost?—*A St. John's Nurse.*

Apply to Messrs. W. H. Bailey and Son, 38 Oxford St., W.

*Mexico.*

(136) Is there an opening for English nurses in Mexico?—*M. M. L.*

Not a good opening, we should say, and we cannot give you the name of a Mexican hospital under English management.

*A Lady Health Inspector.*

(137) Where can I train to become a health inspector?—*Denbigh.*

Write to the Royal Sanitary Institute, Margaret Street, W.

*Employment Wanted for a Man.*

(138) Can you tell me of a Society or anyone who would give light outdoor employment to nurse's husband, out of work through lead poisoning?—*Nurse.*

You might apply to the Church Army, Edgware Road, W., or advertise.

*A Question of Notice.*

(139) A nurse was engaged as matron in charge of a cottage hospital at £30 a year. She is paid her salary quarterly. No express term of leaving was mentioned, but she has been in charge for over two years. What notice ought she to receive or give? Does she come under the domestic service term of a month's notice, or is it legally a hiring for a year?—*Anxious Matron.*

A quarter's notice at least can be claimed on either side.

**Handbooks for Nurses.**

| | Post Free. |
|---|---|
| "A Handbook for Nurses." (Dr. J. K. Watson) ... | 5s. 4d. |
| "Nurses' Pronouncing Dictionary of Medical Terms" | 2s. 0d. |
| "Art of Massage." (Creighton Hale.) ... ... ... | 6s. 0d. |
| "Surgical Bandaging and Dressings." (Johnson Smith.) ... ... ... ... ... ... | 2s. 0d. |
| "Hints on Tropical Fevers." (Sister Pollard.) ... | 1s. 8d. |

Of all booksellers or of The Scientific Press, Limited, 28 & 29 Southampton Street, Strand, London, W.C.

### For Reading to the Sick.

ON THE ANGELS—(THEIR PRESENCE).

In the hours of morn and even,
In the noon and night,
Trooping down they come from heaven
In their noiseless flight
To guide, to guard, to warn, to cheer us
'Mid our joys and cares,
All unseen are hovering near us
Angels unawares.

O faint hearts! what consolation
For us here below,
That angelic ministration
Guides us where we go;
Every task that is before us
Some blest spirit shares;
Watchful eyes are ever o'er us,
Angels unawares.

*J. F. Waller.*

We are "compassed about with a great cloud of witnesses." A great multitude whom no man can number—nearer to us than human language is able to describe, hidden from us by only a thin veil which at any moment may be rent asunder—are silently imploring us, as it were, to rise on the wings of a heaven-inspired faith and hope; to be followers of them, even as they also are of Christ.—*Bishop G. H. Wilkinson.*

As angels were appointed to watch over the saints of God, so did they especially wait on Him Who was the King of saints. They rejoiced at His birth; ministered to Him in His temptation, and in His agony; they proclaimed His resurrection; and hereafter they will attend upon Him, when He shall return in His glory. Thus are these unseen beings continually employed to succour and defend the people of God. They are ever hurrying to and fro with messages of mercy from the courts of God in heaven to the abodes of men on earth. Whether men's eyes are open to discern them, or whether, as is now usually the case, they are withheld from our sight, still they are ever present, ministering in the Church of Christ, and waiting on His members. They are all around us and about us, watching us in our heavenward path, rejoicing at our repentance, and grieving over our falls. They assist us in the trials, and temptations, and duties of life, and are sent to comfort and sustain us in the hour of death.—*Dean Hook.*

A beautiful scrap of instruction out of old rabbinical lore tells us that there are in heaven two kinds of angels—the angels of service and the angels of praise. The latter are of a higher order than the former. No one of them praises God twice, but having once lifted up his voice in the song of heaven, he ceases to be. He has perfected his being. His song is the full flower and perfect fruit of his life, that for which he was made. He has now finished his work, and his life is breathed out in his one holy psalm. There is in this delightful fancy a deep truth, that the highest act of which an immortal life is capable is praise. The unpraising life has not yet realised its holiest mission. It has not yet borne the sweetest, ripest, best fruit, that which in God's sight is most precious of all. In heaven all life is praise, and we come near heaven's spirit only as we learn to praise.—*Dr. J. R. Miller.*

## Figures and Tables

**Figure f1:**